# Effect of Antibacterial Plant Extracts on the Morphology of Electrospun Poly(Lactic Acid) Fibres

**DOI:** 10.3390/ma11060923

**Published:** 2018-05-30

**Authors:** Peiwen Wang, Elisa Mele

**Affiliations:** Department of Materials, Loughborough University, Loughborough LE11 3TU, UK; peiwenwang1992@gmail.com

**Keywords:** electrospinning, phase separation, plant extracts, poly(lactic acid)

## Abstract

Essential oils (EOs) of clary sage and black pepper induce changes in the morphology of poly(lactic acid) (PLA) electrospun fibres. The chemical composition of the oils is analysed by gas chromatography-mass spectrometry and Fourier-transform infrared spectroscopy; while the evaporation rate of the EOs and their main chemical components is characterised by Thermogravimetric Analysis. The addition of EOs generate thermodynamic instabilities during the electrospinning process, leading to the formation of fibres with either wrinkled (for clary sage oil) or nano-textured surfaces (for black pepper oil). The morphology of the PLA-EOs fibres is investigated by Scanning Electron Microscopy. Together with a well-defined structure, the fibres produced also possess antibacterial activity, as demonstrated by viability loss tests conducted on *E. coli* and *S. epidermidis*. Bacteria inactivation efficiency of 76 and 100% is reported for the composite PLA/essential oils electrospun mats. The composite mats produced are promising in the biomedical field, where nanotopography offers physical cues to regulate cell behaviour, and the delivery of therapeutic compounds (essential oils) limits microbial growth.

## 1. Introduction

Essential oils (EOs) are usually extracted from aromatic plants by steam distillation and they are natural complex mixtures of many different low molecular weight compounds, such as terpenoids and phenylpropanoids [1,2]. EOs are widely used in food preservation, agriculture and pharmaceutics, due to their anti-bacterial, anti-fungal, anti-oxidation and anti-inflammation properties [3,4]. In recent years, the encapsulation of essential oils into polymeric nanofibres produced by electrospinning has gained increasing interests, because of the possibility of achieving a longer and more controlled release of those bioactive agents if compared with other delivery methods [5].

Electrospinning is an electro-hydrodynamic process that allows the extrusion of polymer fibres by the application of an electric force [6]. By adjusting the operational parameters and the rheological properties of the material to be spun, fibres with desired size, chemistry and surface properties (fibres decorated with pores, papillae or knots) can be produced. It has been demonstrated that electrospun mats containing essential oils, such as cinnamon [7,8,9], tea tree [10], thyme [11] and lavender [12,13], are promising as advanced wound dressings and smart food packaging systems. Rieger et al. have demonstrated the electrospinning of cinnamon EO in a chitosan matrix, obtaining nanofibres with an inactivation rate higher than 70% for bacteria responsible for skin infections (*Escherichia coli* and *Pseudomonas aeruginosa*) [9]. T. Uyar and collaborators have proposed nanofibres that incorporate cyclodextrin inclusions (CD-ICs) of volatile compounds from essential oils, such as linalool and thymol, for their antibacterial efficiency against *E. coli* and *Staphylococcus aureus* [14,15]. Lavender and *Syzygium aromaticum* (clove) oils have been encapsulated in polyacrylonitrile nanofibers [13,16]. The composite fibres were cytocompatible (high viability of fibroblast cells) and effective against Gram positive and Gram negative bacteria (*E. coli*, *S. aureus*, *Bacillus subtilis* and *Klebsiella pneumonia*). Z. Karami et al. have produced electrospun poly(caprolactone) and poly(lactic acid) mats encapsulating herbal thymol (from thyme EO) for the treatment of skin infections induced by *E. coli* and *S. aureus* [11]. In vivo studies on mice showed that the nanofibrous dressings promoted wound closure and enhanced granulation tissue formation and re-epithelialization of the injured tissue. Those and other studies have established that EOs impart bioactivity to electrospun fibres by making them, for example, effective for the treatment of bacterial colonisation and skin inflammations, and for the inhibition of oxidative processes [5]. However, the effects of EOs on the morphology of electrospun mats have not yet been investigated.

Here, the role played by the chemical composition of clary sage and black pepper essential oils on the topography of electrospun poly(lactic acid) fibres is discussed. Particular attention is given to phase separation phenomena happening during the electrospinning process as a consequence of the evaporation of the most volatile components of EOs and their poor miscibility with the polymer. Morphological investigations of the electrospun mats are combined with chemical and thermal analysis of the essential oils, in order to identify the chemicals mainly responsible for the structural changes observed. Antibacterial tests using two model microorganisms confirm that the ability of both clary sage and black pepper EOs to inhibit bacteria growth is not affected by the electrospinning process.

## 2. Materials and Methods

### 2.1. Materials

Poly(lactic acid) (PLA 4060D, molecular weight of 120,000 g/mol, amorphous polymer with an L-lactide content of around 88 wt%) was obtained from Nature Workds LLC. Clary sage essential oil (extracts of *Salvia sclarea*) and black pepper essential oil (extracts of *Piper nigrum*) were obtained from Umber Maitreya Natura (Bolzano, Italy). Acetone, α-pinene (C_10_H_16_, M_w_ = 136 g/mol), β-pinene (C_10_H_16_, M_w_ = 136 g/mol), limonene (C_10_H_16_ M_w_ = 136 g/mol) and β-caryophyllene (C_15_H_24_ M_w_ = 204 g/mol) were purchased from Sigma-Aldrich (Gillingham, United Kingdom). *Escherichia coli* (*E. coli*, K12 DLB MG2 566, Gram-negative bacterium) and *Staphylococcus epidermidis* (*S. epidermidis*, NCTC 1457, Gram-positive bacterium) were obtained from National Collection of Type Cultures (NCTC, Salisbury, UK). Linalyl acetate (C_12_H_20_O_2_, M_w_ = 196 g/mol), Ringer’s solution, LB broth, Miller (powder, BP1426-500) and LB agar (powder, BP1425-2) were purchased from Fisher Scientific (Loughborough, UK).

### 2.2. Electrospinning Process

PLA solutions for electrospinning were prepared by dissolving the polymer in acetone at a concentration of 14% *w*/*v*. Clary sage essential oil (CS-EO) or black pepper essential oil (BP-EO) were then added to the PLA/acetone solutions. Concentration of 5.0, 7.5, 10.0 and 15.0% *v*/*v* of essential oils were used. PLA solutions containing linalyl acetate, α-pinene, limonene or β-caryophyllene were prepared by dissolving 14% *w*/*v* PLA in acetone and then adding 10% *v*/*v* of the chemical compound.

For the electrospinning process, a 1 mL syringe with a 23G needle was filled with the polymer solutions prepared (PLA/acetone or PLA/acetone/EOs) and connected to a syringe pump (New Era Pump System, NE-300, New York, NY, USA). The flow rate was set at 0.7 mL/h. The needle was clamped to the positive electrode of a high voltage power supply (S1500032-0, Linari Engineering s.r.l., Pisa, Italy), generating a voltage of 12 kV, while the ground electrode was connected to an aluminium collector (air gap distance of 15 cm). All experiments were conducted in normal environmental conditions.

### 2.3. Morphological Investigations

The morphology of the electrospun fibres was analysed by Field Emission Gun Scanning Electron Microscopy (FEGSEM, LEO 1530VP, LEO Elektronenmikroskopie GmbH, Oberkochen, Germany). Prior to observation, the fibrous mats were cut into small pieces (0.3 cm × 0.3 cm), stuck on aluminium stubs by carbon adhesive tapes and then coated using a palladium/gold sputter coater for 90 s (Emitech SC7640 Sputter Coater, Polaron, Laughton, United Kingdom) to produce a conductive surface. The average diameter of the fibres was determined by processing the FEGSEM images using ImageJ (Version 1.52c, National Institute of Heath, Bethesda, MD, USA). Up to 60 fibres were analysed for each type of sample.

### 2.4. Differential Scanning Calorimetry

The glass transition temperature (*T_g_*) of the electrospun mats was investigated by differential scanning calorimetry (DSC, TA Instruments Calorimetric Analyser, Elstree, UK) in the temperature range between −20 °C and 100 °C. The samples (5 mg) were tested at a heating/cooling rate of 10 °C/min in three scans: heating, cooling and heating. The onset point of the temperature step change of each sample from the second heating run was obtained as glass transition temperature.

### 2.5. Chemical Characterisation of the Essential Oils

The chemical composition of clary sage and black pepper essential oil was characterised by Gas Chromatography-Mass Spectroscopy (GC-MS, Varian, Santa Clara, CA, USA). The essential oils were diluted 25 times with ethyl acetate and analysed using a Hewlett Packard mass detector and a HP-5MS column (length 30 m, inner diameter 0.25 mm, film thickness 0.25 μm). The injector, GC-MS interphase, ion source and selective mass detector temperature were maintained at 250, 280, 250 and 150 °C. The oven temperature for both oils was programmed as follows: 60 °C (1 min), 60–185 °C (1.5 °C/min), 185 °C (1 min), 185–275 °C (9 °C/min), 275 °C (2 min). Split injection was performed with helium as carrier gas, with a flow rate of 1.1602 mL/min. The split ratio of the column was fixed at 40:1. The pressure of the column was set at 9.4 psi. The components were identified by comparing the mass spectra obtained with mass spectra of standards obtained with the same column.

Fourier Transform Infrared Spectroscopy (FTIR) analysis was carried out using Attenuated Total Reflection Infrared (ATR-IR) technique (FTIR-8400S instrument, Shimadzu, Columbia, DC, USA). The range of scan was 600–2500 cm^−1^ with 64 scans and resolution of 4 cm^−1^.

### 2.6. Thermogravimetric Analysis

The evaporation rate of the pure essential oils and their main components was analysed by Thermogravimetric Analysis (TGA, TA Instruments Q5000IR, Elstree, UK). The mass loss of each sample was recorded for 1200 min at a constant temperature of 30 °C.

### 2.7. Antibacterial Tests

The antibacterial properties of the electrospun mats were tested against two model microorganisms, *E. coli* and *S. epidermidis* using AATCC Test method 100–2004 (viability loss) [17]. First, 2 bottles of LB broth solution (2.5 g LB broth powder was dissolved in 100 mL water) and 300 mL molten LB agar (1.2 g LB agar powder was dissolved in 300 mL water) were prepared and sterilized in autoclave at 121 °C for 15 min. After the temperature of the LB broth solution dropped to room temperature, single bacteria culture of each test strain was transferred to the prepared LB broth using sterilized inoculating loops. The bacteria culture in the broth solution was grown overnight at 37 °C in a rotary shaker (Fisher Scientific, Loughborough, UK). The broth solutions with *E. coli* and *S. epidermidis* bacterial cells were then diluted to 10^3^ cells/mL and 10^4^ cells/mL by sterilized Ringer’s solution, respectively. The molten LB agar was poured into petri dishes and left at room temperature for 30 min to set. 20 mg of electrospun fibrous mats (PLA, PLA/CS-EO and PLA/BP-EO mats) were cut into small pieces and placed at the bottom of glass jars. The jars were filled with 1.5 mL of bacteria dilution (*E. coli* and *S. epidermidis* dilutions) in order to completely cover the electrospun samples. The jars were thoroughly shaken. Next, 30 μL of liquid containing bacteria were taken from the jars, spread onto the freshly prepared agar plates and incubated at 30 °C for 24 h (“0 h contact time” samples). The jars with the remaining bacterial dilutions and the electrospun fibres were incubated at 30 °C for 24 h. After 24 h, 30 μL of the bacterial solution were spread onto the agar plates and incubated at 30 °C for 24 h (“24 h contact time” samples). The colony-forming units (CFU) were counted for “0 h contact time” and “24 h contact time” samples. The loss of bacteria viability was calculated according to:

$$loss\;of\;viability\;\left(\%\right)=\frac{B-A}{B}\times 100\%$$

where *A* is the number of bacteria recovered from the inoculated samples in the jar incubated for 24 h; *B* is the number of bacteria recovered from the inoculated samples in the jar immediately after inoculation (at 0 h contact time). Each test was run in triplicates.

## 3. Results and Discussion

**Figure 1:**
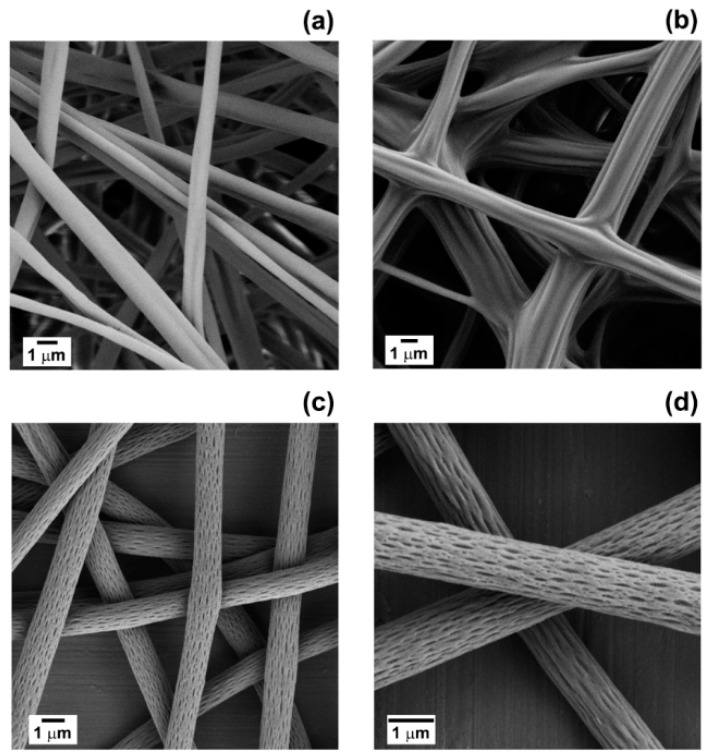
FEGSEM images of electrospun fibres of (**a**) PLA; (**b**) PLA/CS-EO (10% *v*/*v* CS-EO) and (**c**,**d**) PLA/BP-EO (10% *v*/*v* BP-EO) at different magnifications.

Poly(lactic acid) fibres are widely used in biomedical, textile and food packaging sectors, due to their biocompatibility, biodegradability and mechanical properties [18]. PLA electrospun mats can be easily produced from acetone solutions with a polymer concentration ranging from 12.5% to 15.0% *w*/*v* [10,19,20]. For this work, a concentration of 14% *w*/*v* was selected, in order to obtain PLA fibres free from defects and beads and with an average diameter of (1.1 ± 0.5) μm (Figure 1a). As shown in Figure 1b, the addition of 10% *v*/*v* of clary sage essential oil to the PLA/acetone solution (PLA/CS-EO) resulted in highly interconnected fibres with a wrinkled surface. The formation of junctions between fibres and the inter-fibre bonding was attributed to the low glass transition temperature of the composite fibres and to the chemical composition of clary sage EO (as it will be discussed later) [10]. DSC investigations indicated a reduction of *T_g_*. Values of *T_g_* of (15 ± 3) °C and (52 ± 2) °C were measured for PLA/CS-EO and PLA fibres, respectively (DSC graphs are shown in the Supplementary Data, Figure S1). This determined fibre coalescence and fusion at the fibre-fibre junctions [21,22]. A change in glass transition temperature was detected also for PLA fibres containing 10% *v*/*v* of black pepper essential oil (PLA/BP-EO), *T_g_* of (31 ± 3) °C, but no fusion between fibres was observed (DSC graphs are shown in the Supplementary Data, Figure S1). PLA/BP-EO fibres were characterised by a cylindrical shape with average diameter of (1.4 ± 0.2) μm (Figure 1c). The presence of black pepper EO was responsible for the emergence of nano-pores and nano-features onto the surfaces of the fibres (Figure 1d). Each single PLA/BP-EO fibre was covered with pores elongated along the main axis of the fibre, which is the direction of polymer stretching during the electrospinning process. An average of (17 ± 3) pores per μm^2^ of fibre’s area was measured. It was observed that the density of the surface nano-features varied with the amount of EO used and that a concentration of 10% *v*/*v* of BP-EO was suitable to obtain a uniform distribution of well-defined pores (Figure S2 of the Supplementary Data).

**Figure 2:**
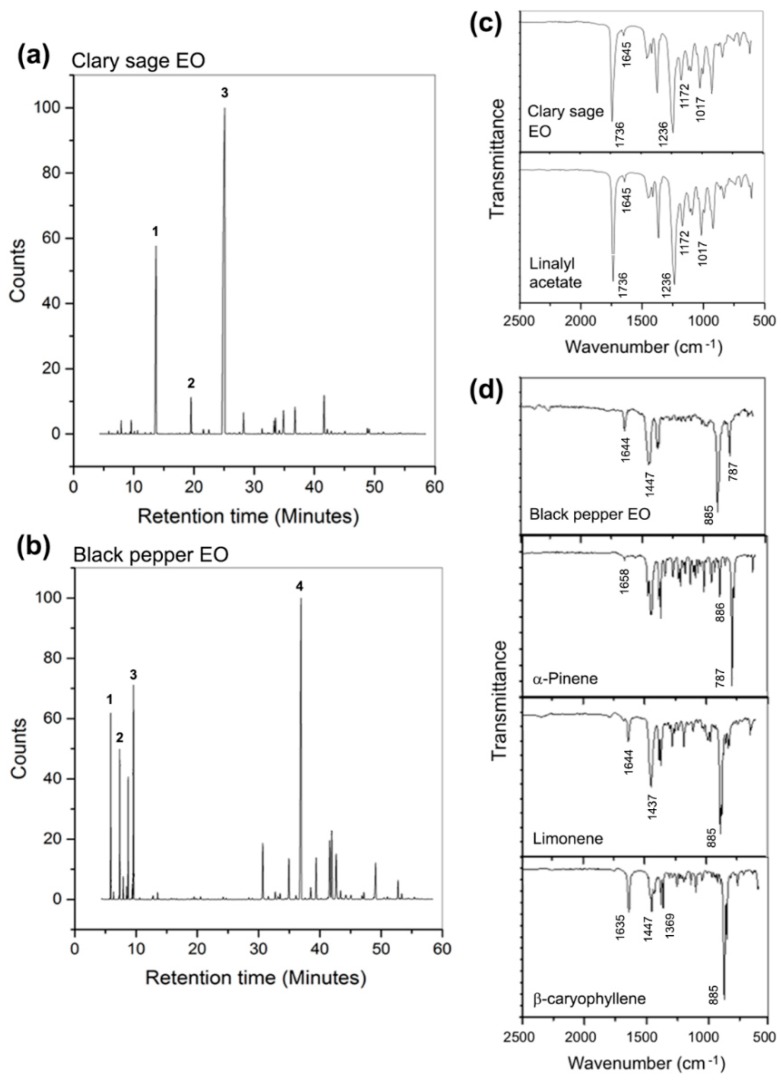
(**a**) Chromatogram of clary sage EO, where 1: linalool, 2: terpineol, 3: linalyl acetate; (**b**) Chromatogram of black pepper EO, where 1: α-pinene, 2: β-pinene, 3: limonene, 4: β-caryophyllene; (**c**) FTIR spectra of pure clary sage EO and linalyl acetate; (**d**) FTIR spectra of pure black pepper EO, α-pinene, limonene and β-caryophyllene.

In order to discuss the role that the chemical composition of clary sage and black pepper EOs played on the morphology of the fibres, GC-MS and FTIR analyses of the essential oils were conducted. GC-MS was used to identify the main chemical components of each EO, since they can vary across different regions of extraction. Figure 2a,b show the chromatograms and the assigned main constituents of clary sage EO (*Salvia sclarea*) and black pepper EO (*Piper nigrum*), respectively. Linalool, terpineol and linalyl acetate were detected for clary sage EO, with retention times of 13.68, 19.51 and 24.95 min, respectively [23,24]. Intense peaks at retention times of 5.85, 7.28, 9.56 and 36.77 min were, instead, identified for black pepper EO, corresponding to α-pinene, β-pinene, limonene and β-caryophyllene, respectively [25,26]. The mass spectra of the main components of clary sage and black pepper EO are shown in Figure S3. FTIR investigations confirmed that the clary sage EO used is indeed rich in linalyl acetate, whose characteristic peaks are visible in Figure 2c: *ν*(C=O) at 1736 cm^−1^, *ν*(C=C) at 1645 cm^−1^ and *ν*(C–O) at 1236, 1172, 1109 and 1016 cm^−1^ [27,28]. The spectrum of black pepper EO has the bands characteristics of α-pinene, limonene and β-Caryophyllene (Figure 2d): *ν*(C=C), *ω*(CH_2_), *ω*(C–H) of α-pinene at 1658, 886, 787 cm^−1^, respectively [29]; *ν*(C=C), *δ*(CH_2_) and *ω*(C–H) of limonene at 1644, 1437 and 885 cm^−1^, respectively [30,31]; *ν*(C=C), *δ*(CH_2_), *δ*(CH_3_) and *ω*(CH_2_) of β-Caryophyllene at 1635, 1447, 1369 and 885 cm^−1^, respectively [29,32].

**Figure 3:**
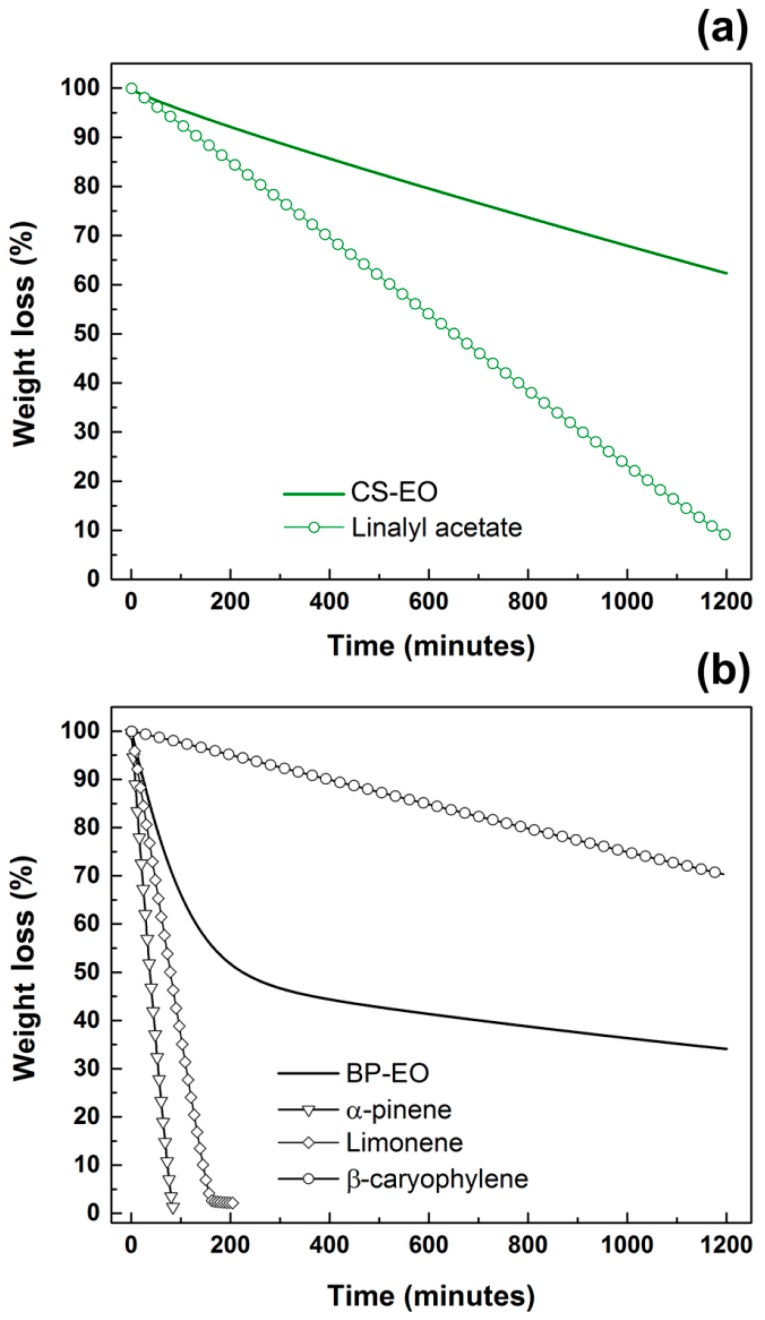
TGA curves of (**a**) clary sage EO and linalyl acetate; (**b**) black pepper EO, α-pinene, limonene and β-caryophyllene.

The chemical compounds identified as main constituents of clary sage and black pepper EOs possess different evaporation rates, which predominantly governed the morphology observed for PLA/CS-EO and PLA/BP-EO fibres [33,34]. The isothermal TGA curves in Figure 3a show that CS-EO had a linear evaporation profile, with a mass loss of circa 40% after 1200 min. A similar linear behaviour was reported for linalyl acetate but with a weight loss of 90% after 1200 min (vapour pressure of 0.005 mmHg at 20 °C). Instead, BP-EO exhibited a two-stage mass loss profile (Figure 3b): 50% weight was lost in the first 200 min; whereas a mass loss of circa 15% was recorded in the remaining 1000 min. The immediate evaporation of BP-EO was correlated to the presence of volatile components. Both α-pinene and limonene evaporated completely in less than 200 min (vapour pressure of 4.5 and 3.3 mmHg at 20 °C, respectively). On the contrary, 70% of β-caryophyllene was still present after 1200 min (0.003 mmHg at 20 °C, respectively).

According to the TGA data, it is expected that, during the electrospinning process, BP-EO will evaporate more quickly than CS-EO, resulting in the formation of highly porous fibres. Studies in the literature have demonstrated that porosity can be generated onto polymer fibres by phase separation phenomena during electrospinning, which are associated with the use of high vapour pressure solvents [35,36,37,38]. Two mechanisms of phase separation are possible during fibres’ spinning: thermally and non-solvent induced phase separation. In the first situation, the fast evaporation of the solvent determines a temperature reduction of the ejected solution, resulting in polymer-rich and polymer-lean regions. After the complete evaporation of the solvent, the polymer-lean domains will originate pores onto the surface of the fibre. Thermodynamic instabilities can also be induced by the addition of a non-solvent to the polymer solution, leading to the formation of polymer-rich and solvent-rich regions. 

**Figure 4:**
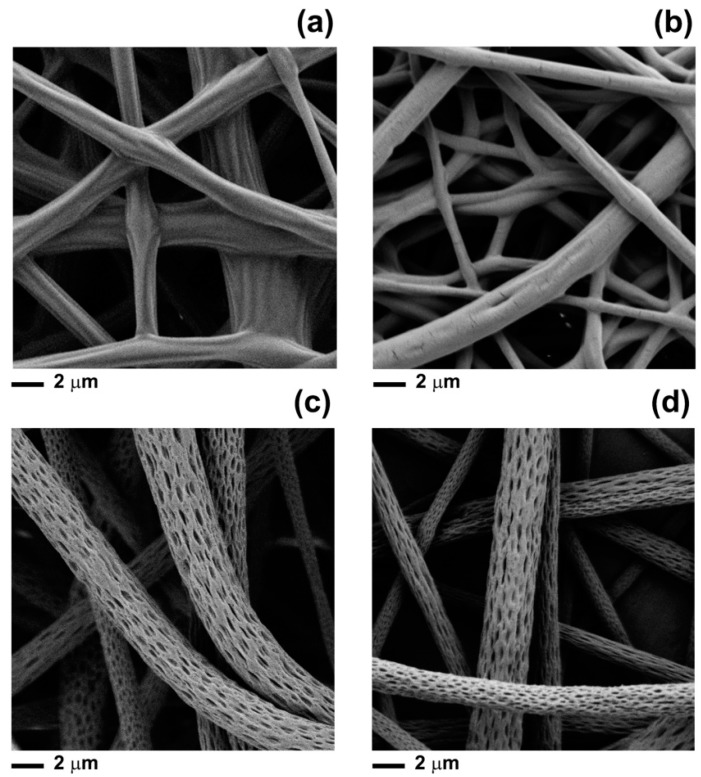
FEGSEM images of fibres electrospun from PLA acetone solutions containing (**a**) linalyl acetate; (**b**) β-caryophillene; (**c**) α-pinene; (**d**) limonene. The concentration of compounds in the starting PLA solution was 10% *v*/*v*.

By considering the Hansen solubility parameters of the chemicals used in this study, only acetone could be regarded as a good solvent for PLA (Table S1 and Figure S4 of the Supplementary Data) [39]. The other compounds (clary sage EO, black pepper EO, linalyl acetate, α-pinene, limonene and β-caryophyllene) were instead non-solvents for PLA. The insolubility of the polymer in the essential oils, together with the diverse evaporation rates of the chemicals constituting the EOs, affected the morphology of the PLA/CS-EO and PLA/BP-EO fibres produced. Fibres electrospun from PLA acetone solutions containing 10% *v*/*v* of linalyl acetate, β-caryophillene, α-pinene and limonene are shown in Figure 4a,b. The morphology of PLA/linalyl acetate and PLA/β-caryophillene mats was very similar to that of PLA/CS-EO fibres (Figure 1b). The surface was either slightly wrinkled (PLA/linalyl acetate) or smooth (PLA/β-caryophillene), and fusion occurred. This morphology can be explained taking into account that, during electrospinning, the rapid evaporation of acetone and the insolubility of PLA in linalyl acetate or β-caryophillene caused thermally- and non-solvent induced phase separation, respectively. Polymer-rich and polymer-lean domains were formed but the slow evaporation of the non-solvents, due to their low vapour pressure, resulted in the fibres being not completely solid when they reached the collector [35]. Consequently, the fibres fused and connected to one another. The effect of the glass transition temperature of the composite fibres was negligible in this case, because *T_g_* of around 45 °C was measured.

PLA/α-pinene (Figure 4d) and PLA/limonene electrospun fibres (Figure 4d) showed surface features recalling those of PLA/BP-EO fibres (Figure 1d), with well-defined and elongated pores uniformly distributed onto their surface. Here, the proposed mechanism of pore formation still includes phase separation events due to rapid evaporation of acetone and non-solubility of PLA in α-pinene and limonene. However, for those mats, the evaporation of the non-solvents transformed the polymer-lean regions in surface pores and significantly increased the concentration of polymer in the ejected filaments [37,40]. The restrained mobility of the polymer chains in the polymer-lean phase prevented the pores from closure. In addition, the stretching of the polymer jet by the electric force caused the elongation of the nano-features.

Since linalyl acetate is the main constituent of clary sage EO, it can be assumed that it played a crucial role in defining the morphology of the PLA/CS-EO mats. For PLA/BP-EO fibres, instead, α-pinene and limonene contributed to the surface porosity much more than β-caryophillene, probably due to their abundance in BP-EO.

**Figure 5:**
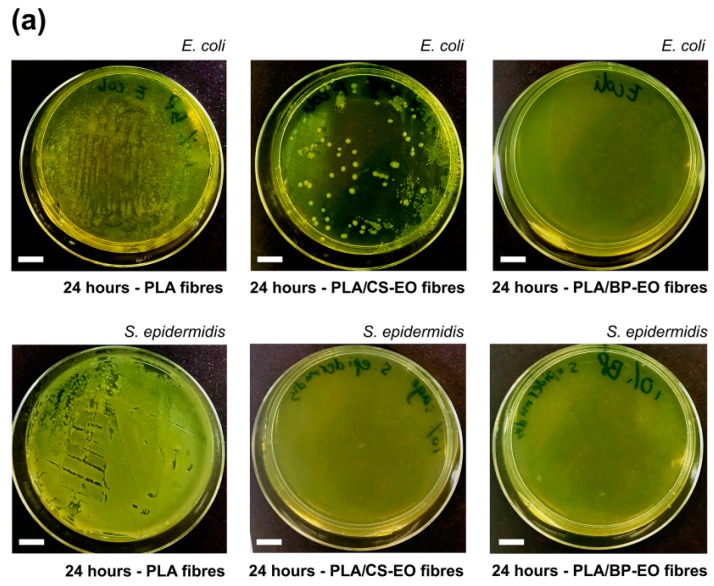

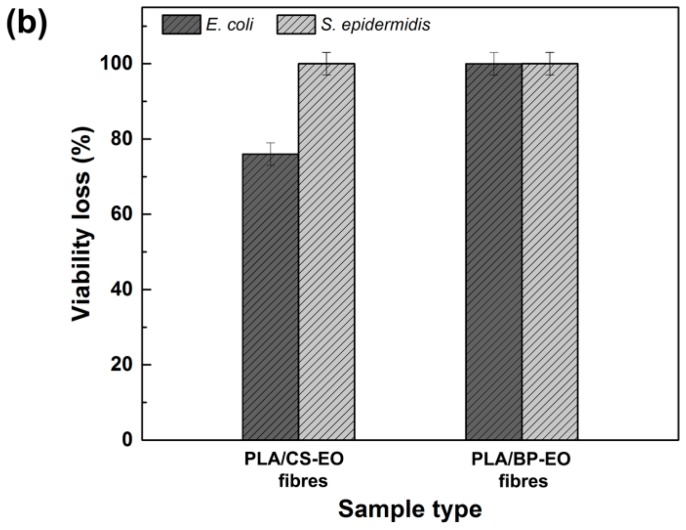
(**a**) Photographs of agar plates showing the growth of *E. coli* (upper row) and *S. epidermidis* (lower row) after 24 h of incubation with PLA, PLA/CS-EO (10% *v*/*v* CS-EO) and PLA/BP-EO electrospun fibres (10% *v*/*v* BP-EO). Scale bar = 1 cm; (**b**) Viability loss of *E. coli* and *S. epidermidis* incubated with PLA/CS-EO and PLA/BP-EO fibres.

The investigations conducted have demonstrated that the complex chemical composition of essential oils can be used to control the morphology of electrospun PLA mats, and nano-textured fibres can be produced by selecting oils rich in volatile compounds, like black pepper EO. Despite the evaporation of some chemical constituents of EOs during electrospinning, both the oils retained their antibacterial activity, as demonstrated by antibacterial tests on two model microorganisms: *E. coli* and *S. epidermidis*. As shown in Figure 5a, after 24 h of incubation at 30 °C, a high number of colony-forming units (CFU) of *E. coli* and *S. epidermidis* were found for PLA fibrous mats without any essential oils. On the contrary, a limited number of *E. coli* colonies and no colonies of *S. epidermidis* (clear agar gel plate) were detected for PLA/CS-EO mats. No growth of *E. coli* and *S. epidermidis* was visible (clear agar gel plates) for PLA/BP-EO fibres. The calculated loss of bacteria viability confirmed that neat PLA mats did not possess antibacterial properties, with inactivation activity of 0% for both microorganisms. Instead, PLA/CS-EO mats were characterised by inactivation activity of 76% for *E. coli* and 100% for *S. epidermidis* (Figure 5b)*.* PLA/BP-EO fibres were highly effective against both bacteria with viability losses of 100%. The limited inactivation efficiency of clary sage EO against *E. coli* can be explained by taking into consideration that Gram-negative bacteria (*E. coli*) are less susceptible to the action of essential oils than Gram-positive ones (*S. epidermidis*). The cell wall of Gram-negative bacteria is characterized by an outer membrane that is formed by diverse proteins and lipopolysaccharides [41]. While small hydrophilic solute can permeate the outer membrane, the diffusion of hydrophobic compounds is limited [4,42]. The structure of the cell wall of Gram-positive bacteria, instead, allows hydrophobic molecules to diffuse inside the cells and affect the action of enzymes and proteins. They can cause disruption of the cell wall with leakage of ions, reduction of membrane potential, depletion of proton gradient and disruption of adenosine triphosphate, eventually resulting in the death of the microorganism [4,41,42].

## 4. Conclusions

Previous studies have established that essential oils impart antibacterial, anti-inflammatory or anti-oxidant properties to electrospun fibres. In this work, medicinal essential oils are used not only to induce bioactivity in electrospun fibres but also to control their structure and surface morphology. Essential oils of clary sage and black pepper have been selected, combined with PLA and processed by electrospinning. The inclusion of essential oils resulted in changes in the chemical, thermal and surface properties of the electrospun fibres, as demonstrated by FT-IR, DSC and SEM investigations. It was demonstrated that during the electrospinning of ternary blends of PLA/acetone/non-solvents (CS-EO, BP-EO and their main components), thermodynamic instabilities and phase separation events were generated with the consequent formation of fibres characterised by wrinkled surfaces or nano-pores. The resulting composite fibres, which also exhibited antibacterial properties, have potential biomedical applications as dressings that are able to prevent bacteria colonisation of wounds and promote skin regeneration. Particularly, the nano-topography created on the surface of the PLA/BP-EO fibres is attractive to the development of scaffolds that provide both physical and chemical cues for skin repair.

## Supplementary Materials

The following are available online at http://www.mdpi.com/1996-1944/11/6/923/s1, Figure S1: DSC curves for (a) PLA, (b) PLA/CS-EO, (c) PLA/BP-EO electrospun fibres, Figure S2: FEGSEM images of PLA/BP-EO fibres containing (a) 5.0% *v*/*v*, (b) 7.5% *v*/*v*, (c) 10.0% *v*/*v* and (d) 15.0 *v*/*v* of BP essential oil, Figure S3: Mass spectra of (a) linalyl acetate, (b) α-pinene, (c) limonene and (d) β-caryophyllene, Figure S4: Hansen space obtained using MATLAB software, and Table S1: Properties of the chemicals used in this study.

## References

[1] Bakkali F., Averbeck S., Averbeck D., Idaomar M. (2008). Biological effects of essential oils—A review. Food Chem. Toxicol..

[2] Raut J.S., Karuppayil S.M. (2014). A status review on the medicinal properties of essential oils. Ind. Crops Prod..

[3] Edris A.E. (2007). Pharmaceutical and therapeutic potentials of essential oils and their individual volatile constituents: A review. Phytother. Res..

[4] Burt S. (2004). Essential oils: Their antibacterial properties and potential applications in foods—A review. Int. J. Food Microbiol..

[5] Zhang W., Ronca S., Mele E. (2017). Electrospun nanofibres containing antimicrobial plant extracts. Nanomaterials.

[6] Mele E. (2016). Electrospinning of natural polymers for advanced wound care: Towards responsive and adaptive dressings. J. Mater. Chem. B.

[7] Liakos I., Rizzello L., Hajiali H., Brunetti V., Carzino R., Pompa P.P., Athanassiou A., Mele E. (2015). Fibrous wound dressings encapsulating essential oils as natural antimicrobial agents. J. Mater. Chem. B.

[8] Wen P., Zhu D.-H., Wu H., Zong M.-H., Jing Y.-R., Han S.-Y. (2016). Encapsulation of cinnamon essential oil in electrospun nanofibrous film for active food packaging. Food Control.

[9] Rieger K.A., Schiffman J.D. (2014). Electrospinning an essential oil: Cinnamaldehyde enhances the antimicrobial efficacy of chitosan/poly(ethylene oxide) nanofibers. Carbohydr. Polym..

[10] Zhang W., Huang C., Kusmartseva O., Thomas N.L., Mele E. (2017). Electrospinning of polylactic acid fibres containing tea tree and manuka oil. React. Funct. Polym..

[11] Karami Z., Rezaeian I., Zahedi P., Abdollahi M. (2013). Preparation and performance evaluations of electrospun poly(ε-caprolactone), poly(lactic acid), and their hybrid (50/50) nanofibrous mats containing thymol as an herbal drug for effective wound healing. J. Appl. Polym. Sci..

[12] Hajiali H., Summa M., Russo D., Armirotti A., Brunetti V., Bertorelli R., Athanassiou A., Mele E. (2016). Alginate-lavender nanofibers with antibacterial and anti-inflammatory activity to effectively promote burn healing. J. Mater. Chem. B.

[13] Balasubramanian K., Kodam K.M. (2014). Encapsulation of therapeutic lavender oil in an electrolyte assisted polyacrylonitrile nanofibres for antibacterial applications. RSC Adv..

[14] Aytac Z., Ipek S., Durgun E., Tekinay T., Uyar T. (2017). Antibacterial electrospun zein nanofibrous web encapsulating thymol/cyclodextrin-inclusion complex for food packaging. Food Chem..

[15] Aytac Z., Yildiz Z.I., Kayaci-Senirmak F., Tekinay T., Uyar T. (2017). Electrospinning of cyclodextrin/linalool-inclusion complex nanofibers: Fast-dissolving nanofibrous web with prolonged release and antibacterial activity. Food Chem..

[16] Yadav R., Balasubramanian K. (2015). Polyacrylonitrile/Syzygium aromaticum hierarchical hydrophilic nanocomposite as a carrier for antibacterial drug delivery systems. RSC Adv..

[17] Madkour A., Tew G. (2008). Towards self-sterilizing medical devices: Controlling infection. Polym. Int..

[18] Gupta B., Revagad N., Hilborn J. (2007). Poly(lactic acid) fiber: An overview. Prog. Polym. Sci..

[19] Mascia L., Su R., Clarke J., Lou Y., Mele E. (2017). Fibres from blends of epoxidized natural rubber and polylactic acid by the electrospinning process: Compatibilization and surface texture. Eur. Polym. J..

[20] Casasola R., Thomas N.L., Trybala A., Georgiadou S. (2014). Electrospun poly lactic acid (PLA) fibres: Effect of different solvent systems on fibre morphology and diameter. Polymer.

[21] Choi S.-S., Lee S.G., Joo C.W., Im S.S., Kim S.H. (2004). Formation of interfiber bonding in electrospun poly(etherimide) nanofiber web. J. Mater. Sci..

[22] You Y., Lee S.W., Lee S.J., Park W.H. (2006). Thermal interfiber bonding of electrospun poly(l-lactic acid) nanofibers. Mater. Lett..

[23] Cai J., Lin P., Zhu X., Su Q. (2006). Comparative analysis of clary sage (*S. sclarea* L.) oil volatiles by GC-FTIR and GC-MS. Food Chem..

[24] Carrubba A., la Torre R., Piccaglia R., Marotti M. (2012). Characterization of an Italian biotype of clary sage (*Salvia sclarea* L.) grown in a semi-arid Mediterranean environment. Flavour Fragr. J..

[25] Singh G., Marimuthu P., Catalan C., De Lampasona M.P. (2004). Chemical, antioxidant and antifungal activities of volatile oil of black pepper and its acetone extract. J. Sci. Food Agric..

[26] Kapoor I.P.S., Singh B., Singh G., De Heluani C.S., De Lampasona M.P., Catalan C.A.N. (2009). Chemistry and in vitro antioxidant activity of volatile oil and oleoresins of black pepper (*Piper nigrum*). J. Agric. Food Chem..

[27] Bombarda I., Dupuy N., Le Van Da J.-P., Gaydou E.M. (2008). Comparative chemometric analyses of geographic origins and compositions of lavandin var. Grosso essential oils by mid infrared spectroscopy and gas chromatography. Anal. Chim. Acta.

[28] Lafhal S., Vanloot P., Bombarda I., Kister J., Dupuy N. (2016). Identification of metabolomic markers of lavender and lavandin essential oils using mid-infrared spectroscopy. Vib. Spectrosc..

[29] Schulz H., Baranska M. (2007). Identification and quantification of valuable plant substances by IR and Raman spectroscopy. Vib. Spectrosc..

[30] Marcuzzo E., Debeaufort F., Sensidoni A., Tat L., Beney L., Hambleton A., Peressini D., Voilley A. (2012). Release behavior and stability of encapsulated D-limonene from emulsion-based edible films. J. Agric. Food Chem..

[31] Ansarifar E., Mohebbi M., Shahidi F., Koocheki A., Ramezanian N. (2017). Novel multilayer microcapsules based on soy protein isolate fibrils and high methoxyl pectin: Production, characterization and release modelling. Int. J. Biol. Macromol..

[32] Schulz H., Ozkan G., Baranska M., Kruger H., Ozcan M. (2005). Characterisation of essential oil plants from Turkey by IR and Raman spectroscopy. Vib. Spectrosc..

[33] Dayal P., Liu J., Kumar S., Kyu T. (2007). Experimental and theoretical investigations of porous structure formation in electrospun fibers. Macromolecules.

[34] Qi Z., Yu H., Chen Y., Zhu M. (2009). Highly porous fibers prepared by electrospinning a ternary system of nonsolvent/solvent/poly(l-lactic acid). Mater. Lett..

[35] Lu P., Xia Y. (2013). Maneuvering the internal porosity and surface morphology of electrospun polystyrene yarns by controlling the solvent and relative humidity. Langmuir.

[36] Natarajan L., New J., Dasari A., Yu S., Manan M.A. (2014). Surface morphology of electrospun PLA fibers: Mechanisms of pore formation. RSC Adv..

[37] Lin J., Ding B., Yu J., Hsieh Y. (2010). Direct fabrication of highly nanoporous polystyrene fibers via electrospinning. ACS Appl. Mater. Interface.

[38] Rezabeigi E., Sta M., Swain M., McDonald J., Demarquette N.R., Drew R.A.L., Wood-Adams P.M. (2017). Electrospinning of porous polylactic acid fibers during nonsolvent induced phase separation. J. Appl. Polym. Sci..

[39] Miller-Chou B.A., Koenig J.L. (2003). A review of polymer dissolution. Prog. Polym. Sci..

[40] Katsogiannis K.A.G., Vladisavljevic G.T., Georgiadou S. (2015). Porous electrospun polycaprolactone (PCL) fibres by phase separation. Eur. Polym. J..

[41] Nazzaro F., Fratianni F., De Martino L., Coppola R., De Feo V. (2013). Effect of essential oils on pathogenic bacteria. Pharmaceuticals.

[42] Vaara M. (1992). Agents that increase the permeability of the outer membrane. Microbiol. Rev..

