# Supplementary material for: Effect of Antibacterial Plant Extracts on the Morphology of Electrospun Poly(Lactic Acid) Fibres
Source: Materials (Basel). 2018 May 30;11(6):923. doi: 10.3390/ma11060923 (PMC6025030; doi:10.3390/ma11060923)
Supplement: Supplementary file 1 [file materials-11-00923-s001.pdf]

Materials 2018, 11, 923; doi:10.3390/ma11060923 www.mdpi.com/journal/materials

Effect of Antibacterial Plant Extracts on the Morphology of Electrospun Poly(Lactic Acid) Fibres

Peiwen Wang and Elisa Mele *

Department of Materials, Loughborough University, Loughborough, LE11 3TU, UK; peiwenwang1992@gmail.com
* Correspondence: e.mele2@lboro.ac.uk

Supplementary data

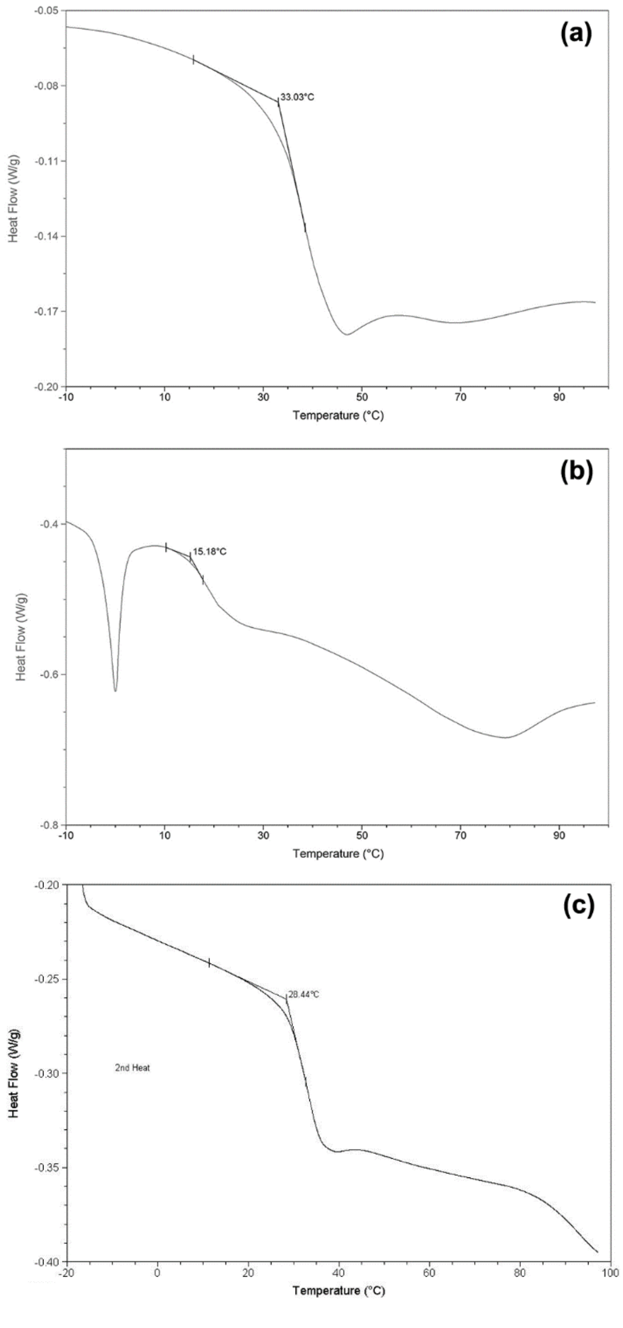

Figure S1: DSC curves for (**a**) PLA, (**b**) PLA/CS-EO, (**c**) PLA/BP-EO electrospun fibres.

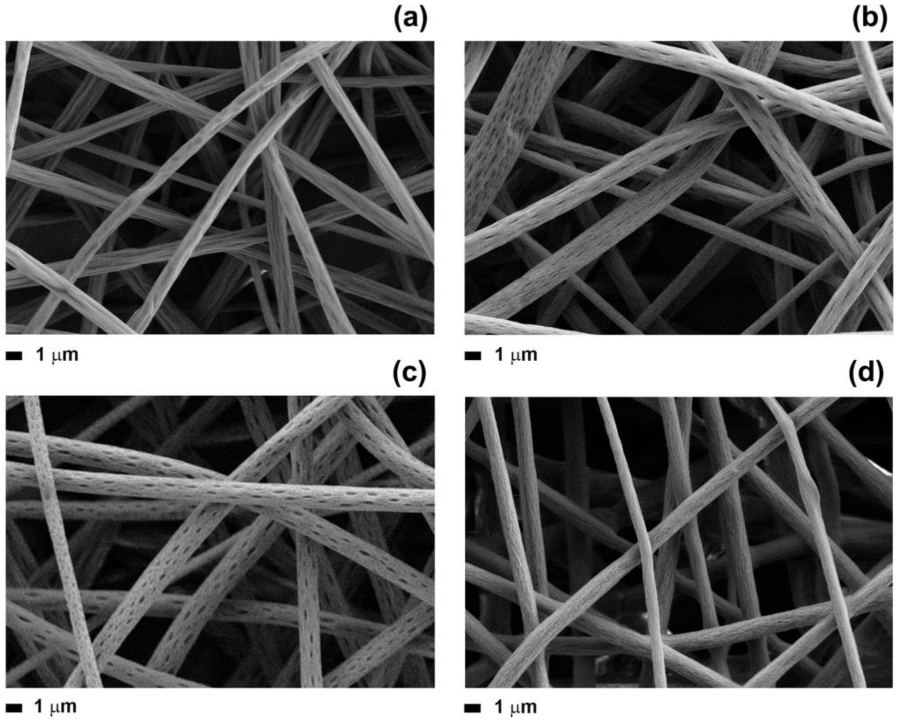

Figure S2. FEGSEM images of PLA/BP-EO fibres containing (**a**) 5.0% *v*/*v*, (**b**) 7.5% *v*/*v*, (**c**) 10.0% *v*/*v* and (**d**) 15.0 *v*/*v* of BP essential oil.

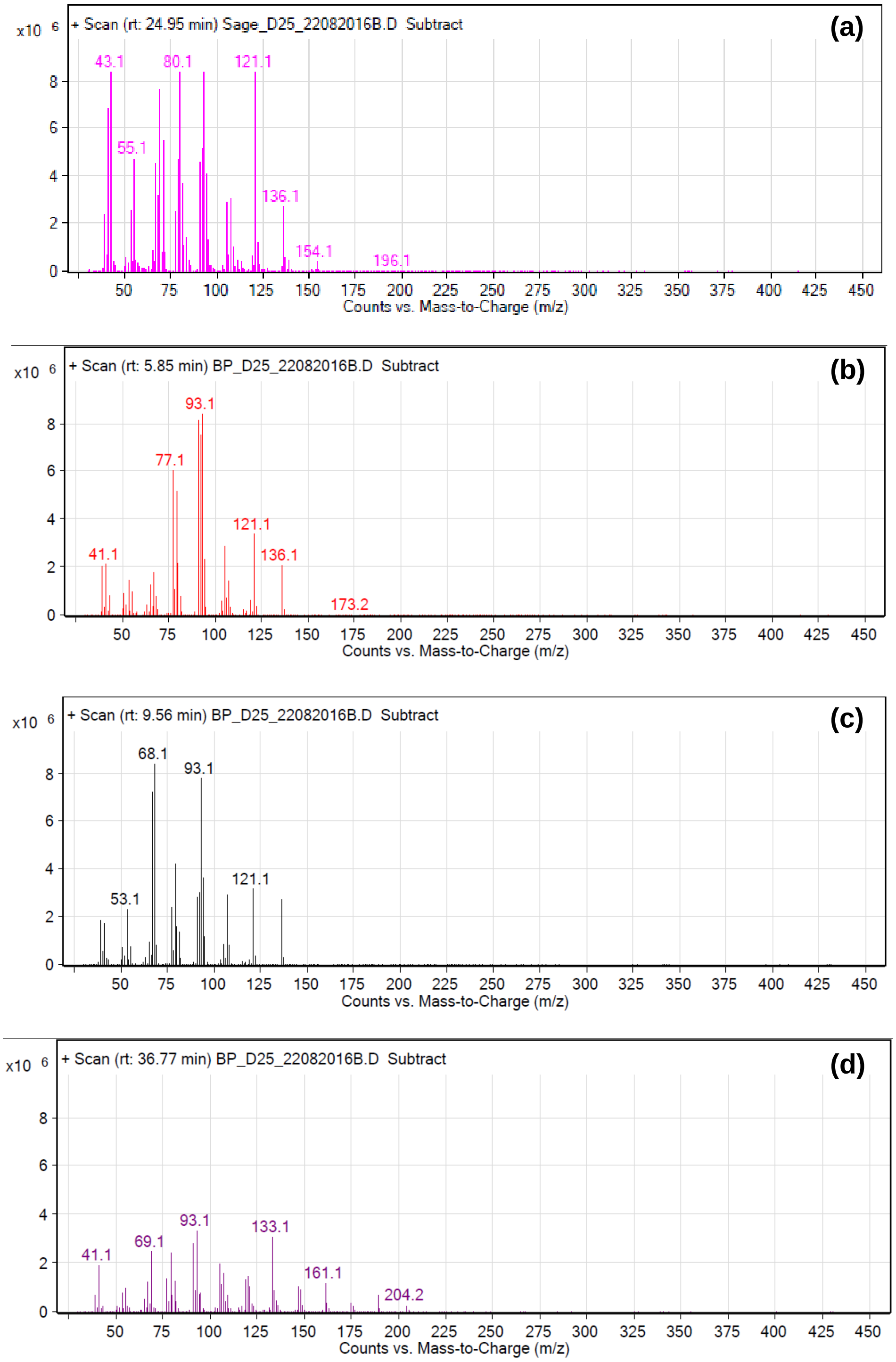

Figure S3. Mass spectra of (**a**) linalyl acetate, (**b**) α-pinene, (**c**) limonene and (**d**) β-caryophyllene.

Table S1. Properties of the chemicals used in this study.

Compound		Vapour Pressure (at 20 °C) [mmHg]	Boiling Point [°C]	Retention Index*	Hansen Solubility Parameter (MPa)[1/2**]			
					δ_d	δ_p	δ_h	δ_t
**PLA**		-	-	-	15.8	8.7	11.1	21.1
**Acetone**		184.000	56	-	15.5	10.4	7.0	19.9
**Black pepper oil**	α-Pinene	4.500	155	0941	17.3	2.4	3.1	17.7
	β-Pinene	2.300	165	0980	17.1	3.0	2.7	17.6
	Limonene	3.300	176	1031	17.2	1.8	4.3	17.8
	β-Caryophyllene	0.003	255	1467	16.9	1.2	3.6	17.3
**Sage oil**	Linalyl acetate	0.005	220	1261	14.3	1.5	6.2	15.6

* Retention Index, also called Kovats retention index, is an index to compare the volatility of materials.
** Hansen solubility parameter (δ_d-dispersion component, δ_p-polar component, δ_h-hydrogen bonding component) can be combined by the equation: ${\delta_t}^2 = {\delta_d}^2 + {\delta_p}^2 + {\delta_h}^2$. The total solubility parameter δ_t, corresponds to the total Hildebrand solubility parameter.

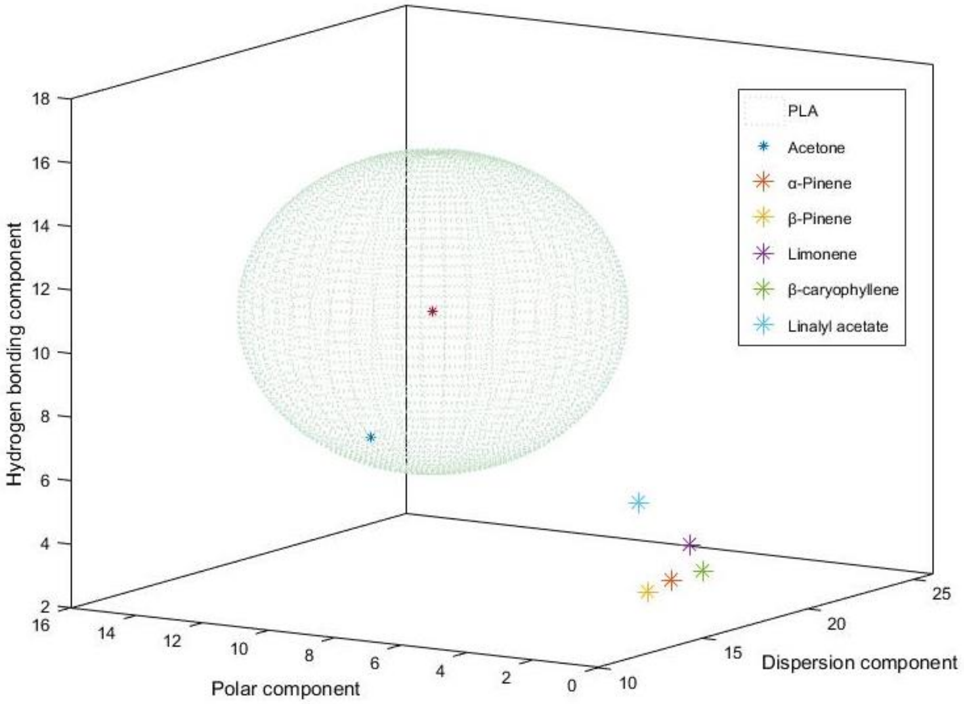

Figure S4. Hansen space obtained using MATLAB software.

Based on the figure, the centre of the sphere is the Hansen solubility parameter of PLA; the sphere is considered as the soluble region of PLA. If the solubility parameter of any solvent is located inside the Hansen sphere, that solvent is a good solvent for PLA; otherwise, the solvent cannot dissolve the polymer.
